# Ship rats and island reptiles: patterns of co-existence in the Mediterranean

**DOI:** 10.7717/peerj.8821

**Published:** 2020-03-19

**Authors:** Daniel Escoriza

**Affiliations:** GRECO, University of Girona, Girona, Girona, Spain

**Keywords:** Alien species, Co-occurrences, Extinction, Island endemic, Lizard

## Abstract

**Background:**

The western Mediterranean archipelagos have a rich endemic fauna, which includes five species of reptiles. Most of these archipelagos were colonized since early historic times by anthropochoric fauna, such as ship rats (*Rattus rattus*). Here, I evaluated the influence of ship rats on the occurrence of island reptiles, including non-endemic species.

**Methodology:**

I analysed a presence-absence database encompassing 159 islands (Balearic Islands, Provence Islands, Corso-Sardinian Islands, Tuscan Archipelago, and Galite) using Bayesian-regularized logistic regression.

**Results:**

The analysis indicated that ship rats do not influence the occurrence of endemic island reptiles, even on small islands. Moreover, *Rattus rattus* co-occurred positively with two species of non-endemic reptiles, including a nocturnal gecko, a guild considered particularly vulnerable to predation by rats. Overall, the analyses showed a very different pattern than that documented in other regions of the globe, possibly attributable to a long history of coexistence.

## Introduction

The Mediterranean basin is a hotspot of biodiversity, but it is also one of the regions in which biodiversity is most threatened, specifically by the massive transformation of landscapes and the spread of alien species (Médail & Quézel, 1999). The loss of biodiversity in the region began in ancient times, shortly after human colonization of the islands (Vigne, 1992). The western Mediterranean archipelagos included rich endemic faunas until the Late Pleistocene, but during 9,000–2,000 years BP they suffered significant impoverishment (Marra, 2013). Most of these extinctions are attributed to human hunting and the spread of introduced continental predators (Vigne, 1992; Marra, 2005). These extinct species were mainly mammals and birds, compared to less affected reptiles, except for large species (Bonfiglio et al., 2002).

The fauna associated with the human colonization of the Mediterranean islands is quite heterogeneous and includes domestic livestock, pets, and small predators that were introduced casually (Gippoliti & Amori, 2006; Bourgeois et al., 2005; Álvarez et al., 2010). One of the most commonly human-transported species is the ship rat, *Rattus rattus*, which even occurs on islets (Masseti, 2012). The presence of this rat on the western Mediterranean islands dates from 2200–2100 years BP (Ruffino & Vidal, 2010), and its expansion was associated with the decline of seabird breeding colonies (Thibault, 1995). The possible effects of these invaders on the Mediterranean island reptiles have not yet been well studied. Rats may have a negative effect on the abundance of some native lizards and geckoes, but this is controversial (Pérez-Mellado et al., 2008; Renet et al., 2013; Corti et al., 2014). However, in the New Zealand archipelago, the presence of alien rats (*R. exulans*) has had a strong negative effect on the occurrence and abundance of island reptiles, forcing some species to extinction (Towns, 1991). Factors explaining such decline of island reptiles facing alien rodents include lack of predator avoidance (Gérard et al., 2014), egg predation, habitat perturbation and trophic competition (Pérez-Mellado & Corti, 1993; Cree, Daugherty & Hay, 1995; Towns, Atkinson & Daugherty, 2006).

At present, the Mediterranean islands still host many endemic reptile species, five of which are found in the western Mediterranean region (Speybroeck et al., 2016). These species occur on both large islands and small satellite islets, except the Gymnesian wall lizard *Podarcis lilfordi* (Speybroeck et al., 2016). This lizard became extinct on the main islands (Mallorca and Menorca) around 3,000 years BP, possibly after the introduction of alien predatory mammals and snakes (Kotsakis, 1981; Pérez-Mellado, Corti & LoCascioa, 1997). These islands are also populated by a cohort of widespread continental reptiles which have reached the western Mediterranean archipelagos through human-mediated dispersals, like the Moorish gecko *Tarentola mauritanica*, the Mediterranean house gecko *Hemidactylus turcicus* and the Italian wall lizard *P. siculus* (Delaugerre & Cheylan, 1992).

Here, I tested whether the presence of *R. rattus* on Mediterranean islands explains that of endemic and non-endemic reptile species. I expected (i) that rats would be negatively associated with the presence of island endemics and some of the non-endemic e.g., semi-fossorial skinks, a guild of reptiles particularly susceptible to predation by rats (Whitaker, 1973). I also expected (ii) that these associations could covary with the size of the islands because predation or competition is particularly intense on islets, compared to larger islands (Holt et al., 1999).

## Materials & Methods

### Study system

The study included 159 islands form the Western Mediterranean region that were grouped as follow: the Balearic Islands (Gymnesian group: Mallorca, Menorca, Cabrera, and satellite islets; Pityusic group: Ibiza, Formentera and satellite islets), Tyrrhenian Islands (Corsica, Sardinia, satellite islets and the Tuscan Archipelago), Provence Islands (Hyères, Lérins, and Riou), and Galite Islands. The largest island included in this study was Sardinia (24,090 km^2^) and the smallest was Rocher de la Folachedda (0.00021 km^2^).

**Figure 1:**
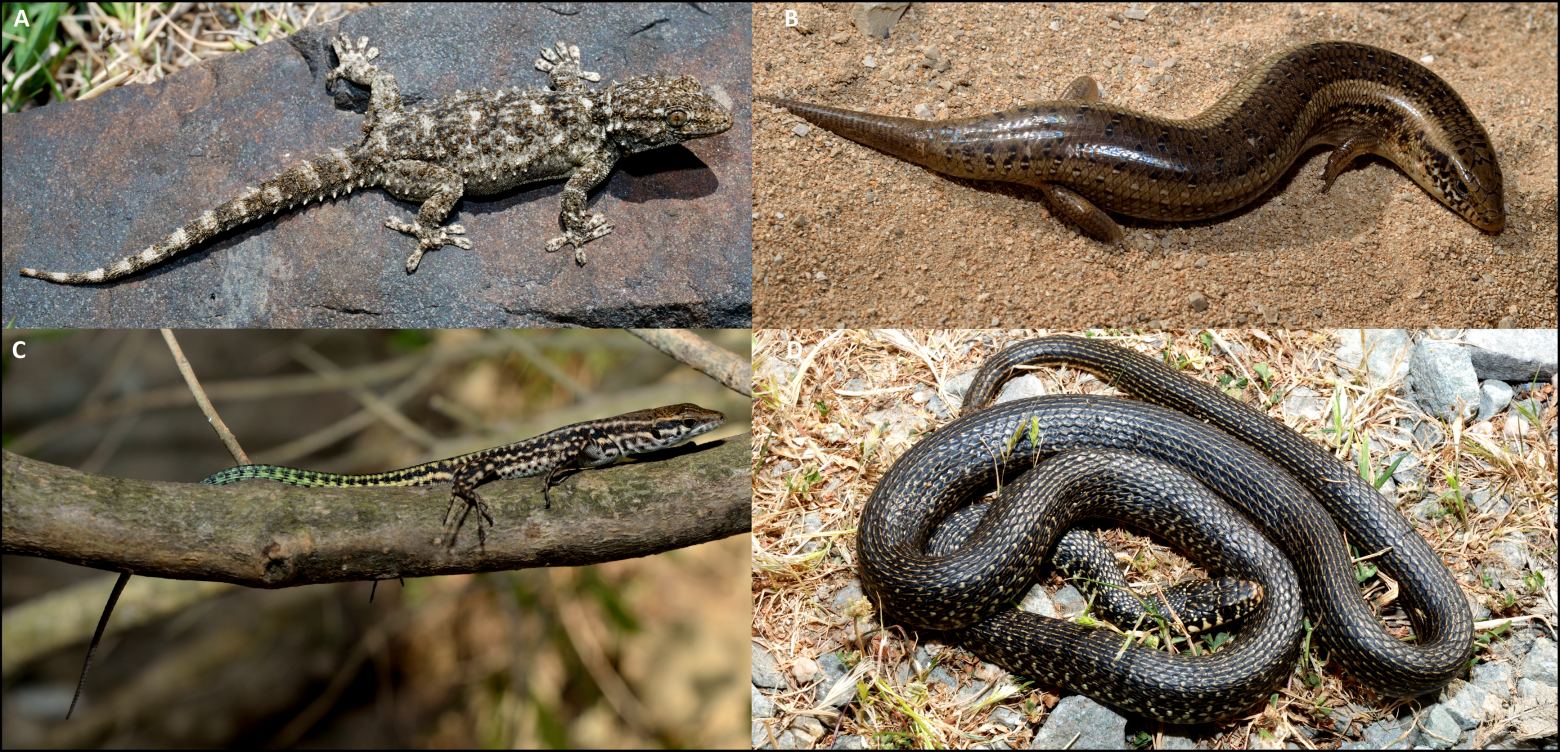
Examples of species and guilds of western Mediterranean island reptiles. (A) Nocturnal gecko: *Tarentola mauritanica*; (B) semi-fossorial skink: *Chalcides ocellatus*; (C) diurnal lizard: *Podarcis tiliguerta*; (D) diurnal snake: *Hierophis viridiflavus*. Photo credits: Daniel Escoriza.

The Balearic Islands include two endemic lizards: *P. lilfordi* in the Gymnesian group and the Ibiza wall lizard, *P. pityusensis*, in the Pityusic group (Mayol, 1997). The Tyrrhenian Islands (Corsica and Sardinia) contain three endemic reptile species: Fitzinger’s algyroides *Algyroides fitzingeri*, Bedriaga’s rock lizard *Archaeolacerta bedriagae*, and the Tyrrhenian wall lizard *P. tiliguerta* (Fig. 1) (Delaugerre & Cheylan, 1992; Sindaco et al., 2006). These occur on the two main islands and satellites, but not on the Tuscan Archipelago. The European leaf-toed gecko, *Euleptes europaea*, is not strictly an endemic island species, although most of its populations occur on the islands of the Corso-Sardinian group, Tuscan Archipelago, Provence and Galite Islands. Although there are also some scattered populations in the coast of mainland Provence, Liguria, and Tuscany (Delaugerre, 2012), in this study *E. europaea* was included in the group of island endemics.

**Figure 2:**
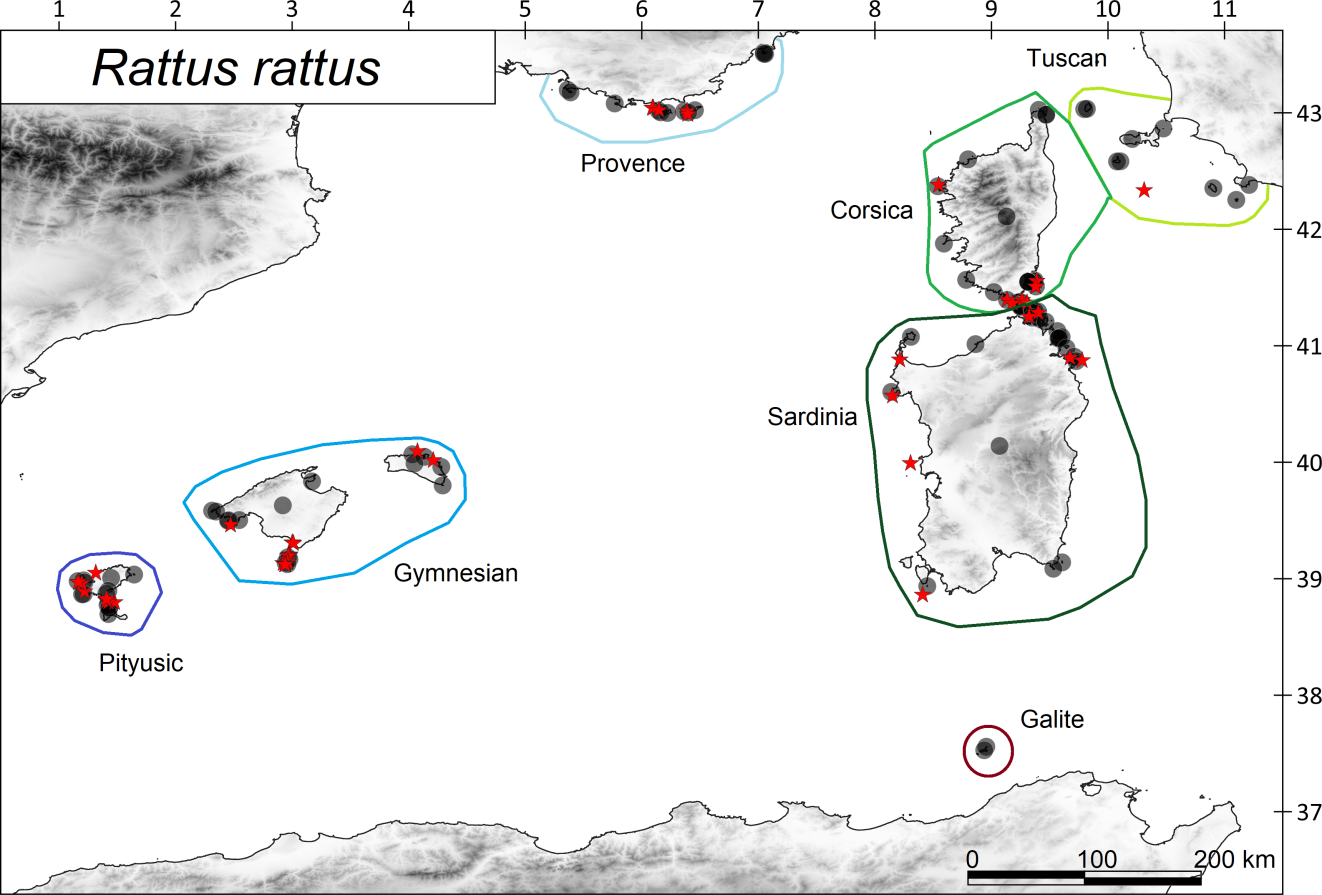
Distribution of the ship rat *Rattus rattus* in the western Mediterranean archipelagos. Islands with presence of rats are shown with filled circles and islands without rats with red starts. The coloured polygons show the island subregions.

**Figure 3:**
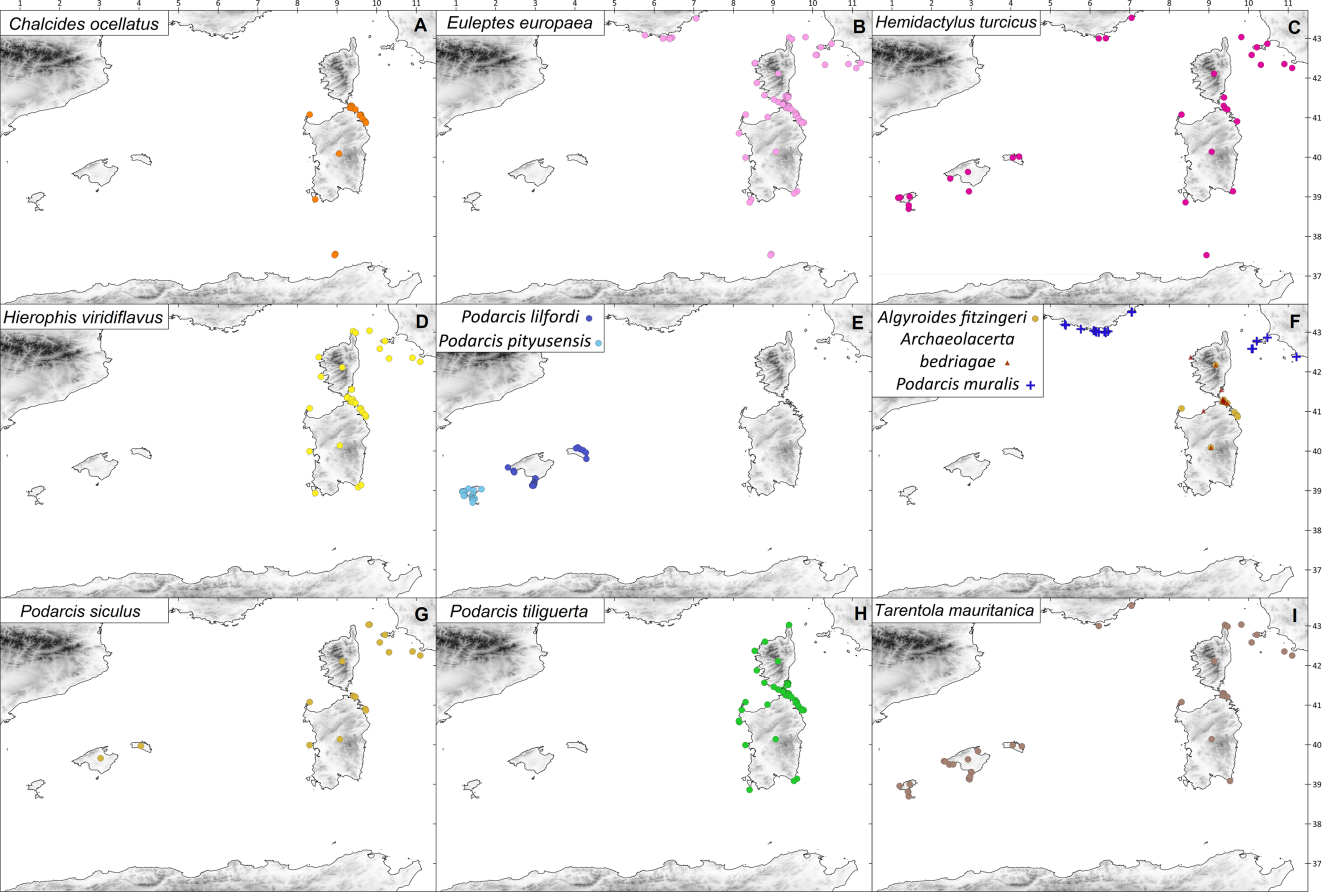
Distribution of the studied reptile species on the western Mediterranean islands. Coloured symbols indicate species presence. (A) *C. ocellatus*; (B) *E. europaea*; (C) *H . turcicus*; (D) *H. viridiflavus*; (E) *P. lilfordi* (navy blue), *P. pityusensis* (sky blue); (F) *A. fitzingeri* (orange), *A. bedriagae* (red), *P. muralis* (blue); (G) *P. siculus*; (H) *P. tiliguerta*; (I) *T. mauritanica*.

The ship rat is the only terrestrial mammal that is distributed throughout the many satellite islets of these archipelagos (Fig. 2; Martin, Thibault & Bretagnolle, 2000). On these islands, there are also some species of continental reptiles, including *Chalcides ocellatus*, *H. turcicus*, *H. viridiflavus*, *P. muralis*, *P. siculus*, and *T. mauritanica* (Fig. 3) (Speybroeck et al., 2016). Other reptile species were not considered in the analysis because the number of islands on which they occur is too small to obtain statistically reliable results. Data on the species’ distributions were obtained from biogeographic atlases and scientific publications: mainly from Cheylan (1983), Delaugerre & Cheylan (1992), Mayol (1997) and Sindaco et al. (2006), and subsequent revisions of the island inventories: Astruc, Couturier & Cheylan (2009), Delaugerre & Ouni (2009), Pinya & Carretero (2011), Corti et al. (2014), Cheylan (2016) and Cheylan, Rivière & Cheylan (2018).

### Data analysis

I evaluated whether there were significant differences between two groups of reptiles (endemic *vs* non-endemic) in the proportion of islands where they coexist with rats, relative to the total number of islands occupied by a given reptile species. Additionally, I investigated whether there are also differences comparing the group of predominantly nocturnal (geckoes) or partially nocturnal species (*C. ocellatus* and *H. viridiflavus*) (Schleich, Kästle & Kabisch, 1996; Delaugerre, 2013) and the diurnal species. In the former groups of species, a greater impact of the rats is expected, because rats mainly forage at night (Towns, 1991). These comparisons were conducted using a Chi-squared test (Pearson, 1900).

I analysed the co-occurrences between species based on binary presence-absence matrices (Gotelli, 2000). In this approach, statistical significance can be computed by comparing the observed co-occurrences with those calculated for randomly generated matrices (Gotelli & Ellison, 2002). However, the capacity of null models to determine associations between species has been questioned, and Markov networks or generalized linear models (GLMs) have been proposed as alternatives (Harris, 2016). In this study, I used logistic regression with Bayesian regularization (Li et al., 2018), with a weakly informative prior, scaled for logistic models (Gelman et al., 2008). In the logistic model, the occurrence of one species was fitted as a response to the occurrence of the other species (i.e., species_1_ = f(species_2_) and species_2_ = f(species_1_)), generating two regression coefficients which were averaged, after testing their correlations (Harris, 2016). I also evaluated the effect of the log(area) of the island on the co-occurrences of species, included as an interacting term in the GLM model e.g., (in R script): species_1_∼rat*log(area). I corrected the *P* values for multiple testing using Holm’s method (Holm, 1979). Model fit was assessed by the Hosmer-Lemeshow goodness of fit test (Hosmer & Lemeshow, 1989). Bayesian GLMs were applied with the arm package (Gelman et al., 2018) and tests of model fit with the ResourceSelection package (Lele, Keim & Solymos, 2019) in the R environment (R Core Development Team, 2019).

## Results

In total, I evaluated the co-occurrences of 12 species of reptiles, six endemics (as *E. europaea* was included in this group) and six non-endemic (Table 1 and Fig. 3). The results of the Chi-squared test showed that of the total number of islands that each reptile species occupies, non-endemic reptiles coexist with rats in a greater proportion of islands: mean endemic = 76.33%, mean non-endemic = 87.32%, *χ*^2^ = 4.199, *P* = 0.0404. However no significant differences were found when comparing these proportions between the nocturnal (mean = 87.54%) and diurnal guilds (mean = 77.74%), *χ*^2^ = 3.551, *P* = 0.0595.

**Table 1:** Reptile species of western Mediterranean islands considered in this study.

Species	Status	Guild	*N* islands	%*N* rats
*Algyroides fitzingeri*	Endemic	Diurnal lizard	12	100.0
*Archaeolacerta bedriagae*	Endemic	Diurnal lizard	12	100.0
*Chalcides ocellatus*	Non-endemic	Semi-fossorial skink	19	94.74
*Euleptes europea*	Endemic	Nocturnal gecko	82	79.27
*Hemidactylus turcicus*	Non-endemic	Nocturnal gecko	32	84.38
*Hierophis viridiflavus*	Non-endemic	Diurnal snake	35	94.29
*Podarcis lilfordi*	Endemic	Diurnal lizard	27	40.74
*Podarcis muralis*	Non-endemic	Diurnal lizard	22	77.27
*Podarcis pityusensis*	Endemic	Diurnal lizard	24	58.33
*Podarcis siculus*	Non-endemic	Diurnal lizard	17	88.24
*Podarcis tiliguerta*	Endemic	Diurnal lizard	54	79.63
*Tarentola mauritanica*	Non-endemic	Nocturnal gecko	40	85.00

**Notes.**

*N* islands total number of islands occupied by each reptile species % *N* rats percentage of *N* populated by the ship rat *Rattus rattus*

The analyses showed that most of the pairs were randomly associated (91.7%) (Table 2). Table 2 showed the averaged GLM coefficients obtained by two models, with the presence of rats included as a predictor and as a dependent variable. Pearson’s correlations showed that in all the models two coefficients were highly correlated and their mean is useful to assess interspecific interactions. *Rattus rattus* only showed a statistically significant positive association with the snake *H. viridiflavus* and positive and marginally significant association with the gecko *T. mauritanica* (Table 2). The analyses also indicated that the size of the islands does not modify the interspecific associations (Table 3). Most of the GLMs showed good fitness as indicated by the Hosmer-Lemeshow tests ( File S2), except the model evaluating the association between *R. rattus* and *P. siculus*, including log(area) as an interacting term.

**Table 2:** Results of the GLMs analyses of the effect of ship rats *Rattus rattus* on the occurrence of western Mediterranean islands reptiles. Significant results (*P* ≤ 0.05) after being adjusted for multiple testing are shown with an asterisk.

Status	Species	Coefficient	*P*	*P*-adjusted
Endemic	*Algyroides fitzingeri*	2.31	0.097	0.485
	*Archeolacerta bedriagae*	2.31	0.097	0.485
	*Euleptes europaea*	0.41	0.456	1.0
	*Podarcis lilfordi*	−2.65	0.051	0.306
	*Podarcis pityusensis*	−0.00	1.0	1.0
	*Podarcis tiliguerta*	0.49	0.399	1.0
Non-endemic	*Chalcides ocellatus*	2.12	0.026	0.104
	*Hemidactylus turcicus*	1.01	0.046	0.138
	*Hierophis viridiflavus*	1.90	0.008	0.048*
	*Podarcis muralis*	−0.13	0.905	0.905
	*Podarcis siculus*	1.16	0.107	0.214
	*Tarentola mauritanica*	1.15	0.014	0.070

**Table 3:** Results of the GLMs analyses of the effect of ship rats *Rattus rattus* and island area on the occurrence of western Mediterranean islands reptiles.

Status	Species	Coefficient	*P*	*P*-adjusted
Endemic	*Algyroides fitzingeri*	0.54	0.085	0.510
	*Archaeolacerta bedriagae*	0.16	0.561	1.0
	*Euleptes europaea*	0.16	0.405	1.0
	*Podarcis lilfordi*	0.02	0.953	1.0
	*Podarcis pityusensis*	−0.00	1.0	1.0
	*Podarcis tiliguerta*	0.24	0.337	1.0
Non-endemic	*Chalcides ocellatus*	0.31	0.303	1.0
	*Hemidactylus turcicus*	0.35	0.112	0.672
	*Hierophis viridiflavus*	0.28	0.284	1.0
	*Podarcis muralis*	0.19	0.508	1.0
	*Podarcis siculus*	0.19	0.451	1.0
	*Tarentola mauritanica*	0.20	0.286	1.0

## Discussion

This study evaluated for the first time the effect of the presence of rats on Mediterranean island reptiles, and contrary to expected, the rats appear to have limited impact on them. The ship rat is the most widespread alien mammal in the Mediterranean islands and its role as a predator of lacertids, geckonids, and small snakes is well established (Sciberras & Schembri, 2008). The ship rat is also an efficient colonizer of small islets, and once it reaches an archipelago, there is frequent inter-island dispersal (Cheylan, Granjon & Britton-Davidian, 1998). Both its diet and dispersal capacity induced historical pernicious effects on island reptiles, particularly on isolated oceanic islands (Thibault et al., 2017). In the study region, endemic reptiles occupy a smaller proportion of islands populated by rats than non-endemic reptiles. Given that rats appear to have a limited effect on the occurrence of endemic species, these differences in the occupation of the islands could be attributed to the fact that rats and alien reptiles have been introduced together through human transportations (Corti et al., 2014; Russell & Delaugerre, 2017).

The presence of *R. rattus* does not determine the presence of island lizards, either endemic or non-endemic. In this sense, previous studies reported that recent eradication of *R. rattus* had no apparent benefit on the demographics of some island populations of lizards (Pérez-Mellado et al., 2008; Krebs et al., 2015). The occurrence of the endemic nocturnal gecko (*E. europaea*) was also unaffected by the presence of ships rats. This small gecko responds to the presence of high densities of rats by modifying its foraging behaviour, thereby reducing predation exposure of adults and juveniles (Krebs et al., 2015). The ship rats were positively associated with two non-endemic species (*T. mauritanica* and *H. viridiflavus*) and were not negatively associated with any*.* This is remarkable because some of these species belong to guilds considered to be particularly susceptible to predation by rats, including nocturnal geckoes and semi-fossorial skinks (Towns & Broome, 2003; Parrish, 2005). In this sense, the results also did not show differences in the occurrence of nocturnal reptiles compared to diurnal ones, in those islands populated with rats. The positive association between *R. rattus* and cohorts of non-native species has been also documented in other groups of biota, such as tenebrionid beetles (Palmer & Pons, 1996).

The fact that rats have been introduced since ancient times possibly allowed lizards to develop several mechanisms to reduce the risk of predation. The nine lizard species studied have the capacity for tail autotomy and are saxicolous, benefiting from refuge provided by small rock fissures (Luiselli et al., 2005; Pafilis, Pérez-Mellado & Valakos, 2008). On islands, rats are frequently frugivorous and widely use trees as foraging ground (Delgado Garcia, 2000), negatively affecting arboreal lizards (Towns, 1991). The geckoes of the Mediterranean islands are mainly lapidicolous species that use trees very occasionally (Schleich, Kästle & Kabisch, 1996; Salvador, 2014), so this type of interaction is possibly of no relevance.

The only species of snake (*H. viridiflavus*) considered in this study showed a positive association with rats. Adults of this species of snake reach sizes of approximately 150 cm and feed exclusively on small vertebrates (Sciberras, 2009; Luiselli et al., 2015). The association between ship rats and *H. viridiflavus* may arise from the predatory role of the snake. For this reason, the control of rat populations is related to the decline of *H. viridiflavus* on some islets (Vanni & Nistri, 2006).

## Conclusions

The results did not indicate that the presence of *R. rattus* negatively influences the reptile occurrence in the western Mediterranean archipelagos. However, this conclusion is supported by analysis of occurrences (presence-absence) and it is possible that although the presence of rats is not associated with complete eradication of a given reptile species, it affects its population status or foraging niche (Hoare et al., 2007). For this reason, the interaction between native Squamata and alien rodents should be also assessed at different temporal and spatial scales and take into account the island peculiarities i.e., environmental heterogeneity, geographical isolation, presence of seabird colonies (Pérez-Mellado et al., 2008; Escoriza, 2020). Further studies should evaluate the potential demographic impact of rats on Mediterranean reptile populations and possible synergistic effects with other alien species.

##  Supplemental Information

10.7717/peerj.8821/supp-1 Supplemental Information 1 Species occurrence data and islands, separated by subregions

10.7717/peerj.8821/supp-2 Supplemental Information 2 Results of the two co-occurrence logistic models (Coef1), and models evaluating the presence of rats interacting with the island size (Coef2) The adjusted P values for multiple testing and model fitness obtained with the Hosmer-Lemeshow tests (HL) are also shown.

## Additional Information and Declarations

### Data Availability

The following information was supplied regarding data availability: Raw data is available as a Supplementary File.
