# Supplementary material for: Ship rats and island reptiles: patterns of co-existence in the Mediterranean
Source: PeerJ. 2020 Mar 19;8:e8821. doi: 10.7717/peerj.8821 (PMC7085892; doi:10.7717/peerj.8821)
Supplement: Supplemental Information 2 — The adjusted P values for multiple testing and model fitness obtained with the Hosmer-Lemeshow tests (HL) are also shown. [file peerj-08-8821-s002.docx]

File S2. Results of the two co-occurrence logistic models (Coef1), and models evaluating the presence of rats interacting with the island size (Coef2). The adjusted P values for multiple testing and model fitness obtained with the Hosmer-Lemeshow tests (HL) are also shown.

| Native | Coef_1_ | P | *HL* | Coef_2_ | P | *HL* |
| --- | --- | --- | --- | --- | --- | --- |
| *Algyroides fitzingeri* | 2.31  2.31 | 0.485  0.485 | 1.0  1.0 | 0.54 | 0.51 | 0.978 |
| *Archaeolacerta bedriagae* | 2.31  2.31 | 0.485  0.485 | 1.0  1.0 | 0.16 | 1.0 | 0.631 |
| *Euleptes europaea* | 0.41  0.41 | 1.0  1.0 | 1.0  1.0 | 0.16 | 1.0 | 0.972 |
| *Podarcis lilfordi* | ‒2.65  ‒2.66 | 0.204  0.204 | 0.99  1.0 | 0.02 | 1.0 | 0.511 |
| *Podarcis pityusensis* | ‒0.00  ‒0.00 | 1.0  1.0 | ‒  ‒ | ‒0.00 | 1.0 | ‒ |
| *Podarcis tiliguerta* | 0.49  0.49 | 1.0  1.0 | 1.0  1.0 | 0.24 | 1.0 | 0.844 |
| Non-native |  |  |  |  |  |  |
| *Chalcides ocellatus* | 2.12  2.12 | 0.076  0.076 | 1.0  1.0 | 0.303 | 1.0 | 0.861 |
| *Hemidactylus turcicus* | 1.01  1.01 | 0.138  0.138 | 1.0  1.0 | 0.35 | 1.0 | 0.457 |
| *Hierophis viridiflavus* | 1.90  1.90 | 0.048*  0.048* | 1.0  1.0 | 0.280 | 1.0 | 0.312 |
| *Podarcis muralis* | ‒0.13  ‒0.13 | 0.905  0.905 | 1.0  1.0 | 0.190 | 1.0 | 0.653 |
| *Podarcis siculus* | 1.16  1.17 | 0.214  0.214 | 1.0  1.0 | 0.190 | 1.0 | 0.018 |
| *Tarentola mauritanica* | 1.15  1.15 | 0.070  0.070 | 1.0  1.0 | 0.200 | 1.0 | 0.183 |
